# Effects of Sexual Attitudes and Alcohol Use on Korean College Students’ Sexual Experience

**Published:** 2017-10

**Authors:** Eun Joo KIM, Sang Suk KIM, Ji Young LIM, Geun Myun KIM

**Affiliations:** 1. Dept. of Nursing, Sangji University, 83 Usan-dong, Wonju-si, Gangwon-do, Republic of Korea; 2. Red Cross College of Nursing, Chung-Ang University, 84 Heukseok-Ro, Dongjak-Gu, Seoul, Republic of Korea; 3. Dept. of Nursing, Inha University, 100 Inha-ro, Nam-gu, Incheon, South Korea; 4. Dept. of Nursing, Gangneung-Wonju National University, 150 Namwon-ro, Heungop-myeon, Wonju-si, Gangwon-do, Korea

## Dear Editor-in-Chief

Recent trends of sexual liberalization and a distorted sex culture have emerged as important social issues regarding the college years. During that period, students are recognized as adults following their completion of education oriented towards the college entrance exam; accordingly, sexual experience becomes more acceptable (1). Therefore, it is necessary to prepare comprehensive approaches and intervention measures that address health, education, and social studies, in order to establish a healthy sexual culture among college students.

The present study examined sexual experience, sexual attitudes, and alcohol use among Korean college students and identified factors affecting students’ sexual experience, in order to provide basic data for use in establishing such interventions.

From June 1 to Sep 30, 2013, 481 college students were recruited from three colleges located in Korea using convenience sampling. A questionnaire was used to examine participants’ sexual experience, sexual attitudes, and alcohol use (1–3). Responses were analyzed using chi-square tests, t-tests, ANOVA, and logistic regression analysis. The institutional review board at Sangji University approved this study prior to commencement (1040782-130924-HR-01-02).

Participants were aged 21.4 ± 1.97 yr; the majority were male (n = 303, 63.1%). Participants’ mean sexual attitude score was 2.91 ± .91. Scores for each subcategory were as follows: permissiveness, 2.53 ± 1.01; sexual pleasure, 2.77 ± .89; sexual responsibility, 4.13 ± .97. Participants’ mean alcohol use score was 10.9; subgroup scores were as follows: 195 participants scored <8 (40.5%), 170 scored 8–15 (35.3%; risky or hazardous drinking), 46 scored 16–19 (9.6%; high-risk or harmful drinking), and 70 scored ≥20 (14.6%; alcohol dependent). The following numbers of participants reported sexual experiences: holding hands: 343 (71.3%); kissing: 319 (66.3%); hugging: 199 (41.3%); caressing (breast and/or genital): 167 (34.7%); sexual intercourse: 201 (41.8%).

Table 1 presents demographics’ association with sexual attitude, alcohol use, and sexual experience. Permissive sexual attitude, sexual pleasure, alcohol use, and male gender predicted sexual experience (Table 2).

**Table 1:** Participant characteristics’ association with sexual attitude, alcohol use, and sexual experience

***Variable***	***Category***	***Sexual attitude***						***Alcohol use***		***Sexual experience***		
		**Permissiveness**		**Sexual pleasure**		**Sexual responsibility**						
		**m (SD)**	**torF (P)**	**m (SD)**	**torF (P)**	**m (SD)**	**torF (P)**	**m (SD)**	**torF (P)**	**Yes (%)**	**No (%)**	**X^2^ (P)**
Gender	Male	19.6 (6.9)	8.13 (<.001)	23.9 (6.8)	7.66 (<.001)	12.1 (3.0)	−3.48 (.001)	11.9 (8.3)	3.90 (<.001)	160 (70.5)	67 (29.5)	−6.95(<.001)
	Female	14.5 (6.1)		19.1 (6.7)		13.0 (2.7)		9.1 (7.6)		41 (32.3)	86 (67.7)	
Grade	Freshman	^a^15.5 (6.6)	9.23 (<.001)	^a^19.6 (6.7)	12.5 (<.001)	^a^ 11.9 (3.4)	2.77 (.041)	10.8 (7.6)	3.32 (.020)	31 (31.3)	68 (68.7)	39.8 (<.001)
	Sophomore	^b^18.1 (6.9)	a<b	^b^22.7 (6.7)	a<b	^b^ 12.6 (2.6)	a<b	10.1 (7.3)		64 (61.5)	40 (38.5)	
	Junior	^b^19.5 (7.2)		^b^24.1 (7.7)		^b^ 12.5 (2.6)		12.6 (9.7)		71 (65.7)	37 (34.3)	
	Senior	^b^19.1 (6.8)		^b^24.2 (5.8)		^b^ 13.0 (2.8)		9.0 (7.6)		35 (79.5)	9 (20.5)	
Residence	Dormitory and rental accommodation	18.3 (7.0)	3.56 (.059)	23.1 (7.1)	6.72 (.010)	12.2 (2.7)	1.34 (.247)	12.6 (8.7)	4.52 (<.001)	103 (58.2)	74 (42.8)	21.66 (<.001)
	With parents or another family	17.1 (7.1)		21.38 (7.1)		12.6 (3.1)		9.3 (7.3)		98 (55.1)	80 (44.9)	

a, b : Post-hoc Scheffe test; 0.05 significance.

**Table 2:** Predictors of sexual experience

***Variables***		***Beta Estimate***	***Standard Errors***	***Wald Test Static***	***P***	***Odds Ratio***	***Nagelkerke R^2^***
Sexual Attitudes	Permissiveness	.113	.029	14.68	.000	1.120	.396
	Sexual pleasure	.118	030	15.14	.000	1.124	
	Sexual	.053	.052	1.06	.304	1.054	
	Responsibility						
AUDIT*		.035	.014	6.29	.012	1.035	.025
Gender (Male = 0, Female = 1)		1.611	.239	45.39	.000	5.009	.173

* AUDIT; Alcohol Use Disorders Identification Test

Male gender, permissive sexual attitude, and high alcohol use positively predicted sexual experience. Male participants showed significantly more open sexual attitudes, more common experience of sexual intercourse, and higher alcohol consumption scores, resembling results from numerous previous studies (4).

In Korea, alcohol use is widely accepted, particularly among males, with alcohol consumption featuring commonly in social and celebratory events and as part of the acclimation process in preparation for one’s working career following graduation. This general acceptance of high alcohol consumption likely increases the risk of problem drinking in the college years, thereby increasing the likelihood of sexual experience. Alcohol consumption and permissive sexual attitudes increased among the higher students’ grades, leading to an increased risk of problem drinking and negative or harmful sexual experiences. This association may partly reflect the lack of a suitable alternative student culture; therefore, interventions aiming to manage problematic sexual activity and alcohol use should aim to convey healthy values and expectations regarding these topics. Therefore, in light of the present findings, interventions aiming to promote Korean college students’ sexual health should target the link between alcohol use and problematic sexual behavior, and identify male students and students holding permissive sexual attitudes as particularly at risk of such behavior and associated negative consequences. Specifically, educators and college administrators should address drinking and sex-related problems by conveying healthy attitudes towards sex using high-accessibility strategies (e.g., cyber-programs (5), parent-participation programs) in order to increase communication with parents and address attitudes towards alcohol.
